# Increased Autonomic Reactivity and Mental Health Difficulties in COVID-19 Survivors: Implications for Medical Providers

**DOI:** 10.3389/fpsyt.2022.830926

**Published:** 2022-05-25

**Authors:** Lourdes P. Dale, Steven P. Cuffe, Jacek Kolacz, Kalie G. Leon, Nadia Bossemeyer Biernacki, Amal Bhullar, Evan J. Nix, Stephen W. Porges

**Affiliations:** ^1^Department of Psychiatry, College of Medicine-Jacksonville, University of Florida, Jacksonville, FL, United States; ^2^Traumatic Stress Research Consortium (TSRC), Kinsey Institute, Indiana University, Bloomington, IN, United States; ^3^Socioneural Physiology Laboratory, Kinsey Institute, Indiana University, Bloomington, IN, United States; ^4^Department of Psychology, University of North Florida, Jacksonville, FL, United States; ^5^Department of Psychiatry, School of Medicine, University of North Carolina at Chapel Hill, Chapel Hill, NC, United States

**Keywords:** COVID-19, autonomic reactivity, adversity, mental health, PTSD, healthcare providers

## Abstract

**Background:**

Because there is a relationship between mental health (MH) and medical adversity and autonomic dysregulation, we hypothesized that individuals infected with COVID-19 would report greater current autonomic reactivity and more MH difficulties (emotional distress, mindfulness difficulties, and posttraumatic stress). We also hypothesized that individuals diagnosed with COVID-19 who are experiencing difficulties related to their prior adversity and those providing medical care to COVID-19 patients would be more negatively impacted due to their increased stress and infection rates.

**Method:**

US participants (*N* = 1,638; 61% female; Age *M* = 46.80) completed online self-report measures of prior adversity, current autonomic reactivity and current MH difficulties, and COVID-19 diagnosis history. Participants diagnosed with COVID-19 (*n* = 98) were more likely to be younger and providing medical care to COVID-19 patients.

**Results:**

Individuals diagnosed with COVID-19 reported increased current autonomic reactivity, being more negatively impacted by their prior MH/medical adversities, and currently experiencing more MH difficulties with an increased likelihood of clinically-significant PTSD and depression (*p* < 0.01 – *p* < 0.001). Current autonomic reactivity mediated 58.9% to 85.2% of the relationship between prior adversity and current MH difficulties; and COVID-19 diagnosis moderated and enhanced the effect of prior adversity on current autonomic reactivity (*p* < 0.01). Being a medical provider was associated with increased current autonomic reactivity (*p* < 0.01), while moderating and enhancing the relationship between current autonomic reactivity and emotional distress and posttraumatic stress symptoms (*p* < 0.05). Combining COVID-19 diagnosis with being a medical provider increased likelihood of clinically-significant PTSD and depression (*p* < 0.01).

**Conclusion:**

Individuals diagnosed with COVID-19, particularly medical providers, have increased current autonomic reactivity that is associated with their prior adversities and current MH difficulties.

## Introduction

The outbreak of the COVID-19 pandemic has placed stress on society that relates to worry of being infected, losing access to necessities and medications, financial instability, and social isolation (1, 2). The potential impacts and effects of these stressors may be explained through polyvagal theory (3). Polyvagal theory suggests there is a neurophysiological framework rooted in human phylogenetic heritage for the body to determine whether an environment is safe. Through the process of neuroception, the autonomic nervous system can detect threats outside of conscious awareness. When in danger, the sympathetic nervous system triggers mobilization (fight or flight) or immobilization (freeze) response to disengage from social interaction. Mobilization may manifest into chronic anxiety or irritability, whereas immobilization may lead to death feigning, syncope, dissociation, withdrawal, loss of purpose, social isolation, and depression (2).

Along with societal stressors that may retune the autonomic nervous system to react to a potential threat, there may be a link between COVID-19 diagnosis and autonomic dysregulation that may relate to the body's reorganization to fight the disease. One study found the COVID-19 patients in the acute and chronic phase experienced tachycardia, labile blood pressure, muscular fatigue, and shortness of breath (4). Given that autonomic dysregulation can also contribute to these symptoms, the author called for testing, research, and interventions that target the autonomic nervous system (ANS). Another study found significant differences in autonomic functioning in severe and mild COVID-19 patients compared to the control group, as indicated by their heart rate and blood pressure variability and lower baroreceptor sensitivity which put them at risk for sudden cardiac death (5). Research also suggests that the changes in the autonomic nervous system may persist after the infection has dissipated (6). Amongst COVID-19 survivors, symptoms include orthostatic hypotension, postural tachycardia syndrome, orthostatic intolerance, and sudomotor, gastrointestinal and pupillomotor dysfunction (6). Thus, the COVID-19 infection may alter the functioning of the autonomic nervous system, suggesting a need to look at how the COVID-19 infection, outside of societal stressors, may relate to autonomic reactivity.

In addition to medical consequences associated with the infection, there have been numerous reported mental health effects such as depression, anxiety, insomnia, and executive functioning and psychomotor difficulties, as well as decreased quality of life (7–14). In a study examining brain scans pre and post COVID-19 infection, the virus was found to be associated with a reduction in gray matter thickness in fronto-parietal and temporal regions of the brain as well as significant cognitive decline, which persisted even when only examining mild cases (15). The effects of the COVID-19 infection also include difficulties with thinking, concentrating, and memory, known as brain fog, which is hypothesized to be a result of infection and inflammation of cells of brain vessels (16). Considering autonomic reactivity is an indicator of overall physical and mental wellbeing (17), it is important to take this into account as it may further explain how the COVID-19 infection may relate to MH difficulties.

A particularly vulnerable group to autonomic dysregulation may be those who have experienced prior adversity, as their ANS may be retuned to be more reactive, and thus more sensitive to future threats. One study found that in uninfected participants during the pandemic increased autonomic reactivity mediated the relationship between prior MH adversity and current MH difficulties that were not medically related (1). However, there is a need to go beyond asking about the occurrence of an event, such as emotional abuse, and to focus on the perceived impact of the experience as it may relate to the frequency and severity of the events. This is important as individuals who are more impacted by their adversity history may experience greater alterations in their ANS.

Medical adversity may also impact autonomic regulation. Changes in autonomic functioning are present in fibromyalgia, which is characterized by chronic, widespread pain and symptoms of fatigue and dizziness (18). Autonomic dysregulation among fibromyalgia patients include hyperactivity at rest (associated with cold extremities, irritable bowel syndrome, interstitial cystitis), hypoactivity during stress (associated with persistent fatigue, low blood pressure, dizziness, and faintness), sleep disruption, and postural orthostatic tachycardia syndrome (POTS) (18, 19). POTS, a common abnormality of the autonomic nervous system frequently diagnosed with fibromyalgia, consists of autonomic failures such as dysregulated blood flow and orthostatic tachycardia (19).

One mechanism for autonomic dysregulation may be through the immune system as the level of activity and responsivity of discharges in the sympathetic and parasympathetic nerves is affected by cytokines and other immune factors (20). This connection between the ANS and the immune response is discussed in gut-microbiome homeostasis (21) and in theories behind the etiology of depression that link elevated pro-inflammatory cytokines with major depressive disorder (22).

The interaction between the immune system and ANS is evident in multiple sclerosis (MS), an autoimmune disease involving dysregulation of both sympathetic and parasympathetic systems (23). The parasympathetic nervous system is largely driven by the vagus nerve (10th cranial nerve) activity that interacts with the acetylcholine receptors in the body and is involved in anti-inflammatory pathways and cellular immune function (23). This is important for MS, as the pathophysiology of this disease involves over-activation of immune cells that begin to attack the body's own cells, producing symptoms including but not limited to pain, fatigue, loss of sensation, difficulty swallowing, and depression. When communication between the ANS and immune system is dysregulated, inflammatory responses may influence the progression of MS activity, which may induce or worsen a flair of MS (23). Thus, it may be that ANS dysfunction contributes to MS disease progression, specifically through changes in communication with the immunological system (23). Similarly, individuals impacted by their prior medical adversities who are infected with the COVID-19 virus may exhibit increased autonomic reactivity because their ANS may be “retuned” to optimize reactivity to threat and consequently experience MH difficulties associated with autonomic dysregulation.

With healthcare providers being the frontline workers during the COVID-19 pandemic, there has been an increasing concern about their mental and physical health. The results of a large systemic review of the literature suggest that medical providers are at risk of reporting MH difficulties such as anxiety, depression, distress, and sleep problems, which may relate to their work demands, COVID-19 exposure, and lack of personal protective equipment (24). A review study found healthcare workers reported these mental health problems in addition to emotional exhaustion, depersonalization, lack of personal accomplishment, and somatic symptoms such as decreased appetite, indigestion, and fatigue (25). COVID-19 work related stressors such as caring for infected patients, witnessing patient deaths, shortages of equipment, and increased professional demands, may also contribute to a decline in their mental health (26, 27). One study found significantly higher rates of anxiety about spreading the virus to loved ones, mental exhaustion, and posttraumatic stress symptoms in healthcare workers placed in the COVID-19 unit compared to healthcare workers in other units (28). Another study found high rates of moral injury in healthcare providers that were related to how much COVID-19 impacted their work life, concerns about COVID-19 protective equipment, and how supported they felt by their administrative leadership (29).

The worries of the medical providers are founded in reality as they have an increased risk of contracting COVID-19 (2,747 cases per 100,000 people) compared to the general population (242 cases per 100,000 people); suggesting the need to look at related outcomes of the diagnosis itself on this population (30). Given the mental health consequences associated with a previous COVID-19 infection in the general population (7–14), one would anticipate similar or more severe outcomes in medical providers. However, little research has been done on how being infected with COVID-19 may influence this population's mental health. One study found that medical workers with a history of a COVID-19 infection had significantly higher prevalence of stress, anxiety, depression, and PTSD compared to medical workers with no COVID-19 infection history (31). Therefore, both the COVID-19 diagnosis and stress that healthcare providers experience may result in increased levels autonomic reactivity that retune their autonomic nervous systems and worsen their mental health.

The current study investigates whether individuals infected with COVID-19 are experiencing higher levels of self-reported current autonomic reactivity and more MH difficulties, and whether their difficulties would relate to their prior MH and medical adversities. Polyvagal theory would suggest that individuals more impacted by their prior adversities would be more vulnerable to and impacted by the COVID-19 virus, and that their COVID-19 infection exacerbating their prior vulnerabilities and leading to more MH difficulties (i.e., emotional distress, mindfulness difficulties, and posttraumatic stress symptoms). Specifically, the increased autonomic reactivity associated with their prior adversity and the COVID-19 infection would be associated with greater negative MH difficulties. Thus, we hypothesized that

- Individuals infected with the COVID-19 virus will report higher levels of current autonomic reactivity and having been more impacted by their prior MH and medical adversities.
- Individuals more impacted by their prior MH and medical adversities will report experiencing higher levels of current autonomic reactivity.
- COVID-19 diagnosis will interact with prior adversity to impact current autonomic reactivity.
- COVID-19 diagnosis will moderate the relationship between prior MH and medical adversity and current MH difficulties, with individuals infected by the COVID-19 virus who are more impacted by their prior adversities reporting more current MH difficulties (i.e., emotional distress, mindfulness difficulties, and PTSD symptoms).
- Increased current autonomic reactivity will mediate the relationship between prior MH and medical adversity and current MH difficulties.

In addition, this study will explore whether medical providers, who are on the frontline of caring for COVID-19 patients and at greater risk of contracting COVID-19, may be more negatively impacted. Specifically, it explores whether they report higher levels of current autonomic reactivity and current MH difficulties, and if their increased current autonomic reactivity relates to their MH difficulties beyond the general population effects reported for COVID-19 diagnosis.

## Method

### Procedure

All procedures performed in studies involving human participants were in accordance with the ethical standards of the institutional and/or national research committee and with the 1964 Helsinki declaration and its later amendments or comparable ethical standards. After receiving Institutional Review Board approval, data collection began on March 29, 2020. Data was collected through fall of 2020, which coincides with the first wave of COVID-19 in the United States. The only inclusion/exclusion criteria was that individuals needed to be 18 years or older. Participants were recruited *via* social media postings on Reddit, Twitter, Facebook, Instagram, and email lists. To increase the percentage of male, low income, and non-Caucasian responders in the U.S., additional individuals were recruited *via* Qualtrics Panels and paid according to their compensation plan (e.g., cash, airline miles). On the study landing page, participants read the consent form and decided whether to participate. The survey data underwent quality analysis *via* automated checks.

### Constructs and Measures

The survey asked participants whether they have been diagnosed with COVID-19 and if they had experienced physical symptoms related to COVID-19. The latter information was used to eliminate participants from the sample who may have had COVID-19 but did not get an official diagnosis, as data collection began at a time when testing for COVID-19 was not readily available. Demographic factors, such as their age, gender, racial identity, and education and income level, were additionally collected. Below is a description of the constructs and measures and the analyses assessing internal consistency of the measures *via* Cronbach alpha (α).

*Current autonomic reactivity* was assessed *via* the Body Perception Questionnaire Short Form (32, 33), a 20-item measure that assesses self-reported experiences of reactivity in organs and tissues regulated by the ANS. The respondent indicates frequency of bodily sensations using a 5-point Likert scale (1 = *never* and 5 = *always*). Higher scores indicate destabilized autonomic reactivity and have been found to relate to lower parasympathetic activity, higher resting heart rate, and less parasympathetic and sympathetic flexibility in response to challenges (Kolacz, Lewis et al., in preparation). This measure has good convergent validity, internal consistency, high test-retest reliability, and consistent factor structure across samples (34–36).

*Mental and medical health history* represents participant's reported diagnostic history and how impacted they were by their mental and medical health experiences. Two questions asked whether they had a medical diagnosis believed to increase their COVID-19 risk (e.g., heart condition, chronic lung disease, moderate to severe asthma) or had a prior psychiatric diagnosis.

Impact of prior MH and medical adversity was assessed *via* a preliminary version of the Adverse and Traumatic Experiences Scale (1, 37). This instrument asks about prior MH adversity (19 items; α = 0.86), which includes caregiver adverse experiences, caregiver maltreatment, non-caregiver maltreatment, life-threatening situations, sudden death of close ones. It also asks about prior medical adversity (6 items; α = 0.78), which includes serious chronic health condition (e.g., diabetes), severe asthma attack that did not respond to medication, life-threatening illness (e.g., cancer), life-threatening injury requiring hospitalization, traumatic brain injury, invasive surgery with general anesthesia. For all the items, the participants indicate how impacted they were *via* a 5-point Likert scale (0 = *event did not occur*, 1 = *occurred and no impact on my life*, 2 = *minimal impact on my life*, 3 = s*ome impact on my life*, and 4 = *big impact on my life*).

### Current Mental Health

We focused on measures assessing emotional distress, mindfulness difficulties, and posttraumatic stress symptoms. In addition, two measures were used to determine if the participants scored above the clinical cutoff for PTSD and depression. The specific measures used are described below:

Emotional distress was measured *via* a 12-item instrument designed to assess extent of distress symptomatology listed in the Center for Disease Control Website. The respondent indicated *via* a 5-point Likert scale (0 = *not at all*, 1 = *a little bit*, 2 = *moderately*, 3 = *quite a bit*, and 4 = *extremely*) if they were experiencing signs of distress (e.g., anger/fear, sadness, bothered by things that did not bother them before, everything feels like an effort, feelings of disbelief, and increased substance use). The internally consistent items (α = 0.92) were combined to form a total score, with higher scores representing greater emotional distress.

Mindfulness difficulties was measured *via* the Mindful Attention Awareness Scale (38), which includes 15-items that assess dispositional mindfulness, such as open and receptive awareness of what is presently occurring. The respondent rates frequency of everyday experiences *via* a 6-point Likert scale (1 = *almost always*, 2 = *very frequently*, 3 = *somewhat frequently*, 4 = *somewhat infrequently*, 5 = *very infrequently*, and 6 = *almost never*). For the current study, the items were reverse scored so that higher total scores reflect higher levels of mindfulness difficulties. This measure has strong psychometric properties (38) and was found to be internally consistent with the current sample (α =0.90).

Posttraumatic stress symptoms were measured using the PTSD Checklist Civilian Version (39), which is a 17-item self-report measure assessing posttraumatic stress symptoms over the past month related to a traumatic event using a five-point Likert-type scale (0 = *not all*, 1 = *a little bit*, 2 = *moderately*, 3 = *quite a bit*, 4 = *extremely*). This measure has good convergent validity, internal and temporal stability, and test-retest reliability (40), and was found to be internally consistent with the current sample (α =0.96). For the current study, we also focused on the categorization of whether participants scored above or below the clinical cutoff, which is reached by endorsing at least one re-experiencing item, three avoidance items, and two hyperarousal items (41).

Depression was assessed *via* the Patient Health Questionnaire-2 (42, 43), which assesses frequency of depressed mood and anhedonia over the past 2 weeks *via* a 4-point Likert-type scale (0 = *not at all*, 1 = *several days*, 2 = *more than half the days*, and 3 = *nearly every day*). The scores for the two items are summed to determine if the respondent meets clinical cutoff (total score is 3 or greater), which suggests the need for further assessment for depressive disorder.

### Statistical Analyses

To assess differences in current autonomic reactivity and prior mental health histories in individuals infected or not infected by COVID-19, ANOVA and chi square analyses compared the groups with regard to their current autonomic reactivity, mental health history (prior diagnosis and impact of MH and medical adversity), and current MH difficulties (emotional distress, mindfulness difficulties, and posttraumatic stress symptoms). To investigate whether individuals more impacted by their prior MH and medical adversities report experiencing higher levels of current autonomic reactivity, linear regression analyses were run. To test the hypothesis that COVID-19 diagnosis interacts with prior adversity to impact current autonomic reactivity, hierarchical linear regression analyses were run. The first model included as predictors the individual and combined impact of prior MH adversity and COVID-19 diagnosis in step 1, and then included medical adversity in step 2 to determine if its inclusion influenced the predictive power of the variables entered in step 1. Similarly, the second model included as predictors the individual and combined impact of prior medical adversity and COVID-19 diagnosis in step 1, and then included MH adversity in step 2 to determine if its inclusion influenced the predictive power of the variables entered in step 1.

Moderated mediation analyses *via* SPSS Process model 7 explored whether autonomic reactivity mediated the relationship between prior adversity and the current MH difficulties, and whether the relationship between prior adversity and current autonomic reactivity was moderated by COVID-19 infection group status and thus was different for the two groups. The hypothesized moderated mediation model (see **Figure 3**) was tested using a bootstrapping approach in multiple models to assess the significance of the indirect effects at the two levels of the moderator (44). Previous MH and medical adversity were the predictor variables, with current autonomic reactivity as the mediator. The outcome variables were current MH difficulties (i.e., mindfulness difficulties, emotional distress, and posttraumatic stress) and COVID-19 diagnosis was the proposed moderator. Moderated mediation analyses test the conditional indirect effect of a moderating variable (i.e., COVID-19 diagnosis) on the relationship between a predictor (i.e., MH or medical adversity) and an outcome variable (i.e., mindfulness difficulties, emotional distress, or posttraumatic stress) *via* potential mediators (i.e., COVID-19 diagnosis). The “PROCESS" macro, model 7, v2.16 (44) in SPSS version 23 with bias-corrected 95% confidence intervals (*n* = 10,000) was used to test the whether the indirect (i.e., mediated) effects were mediated by current autonomic reactivity (i.e., conditional indirect effects). This model explicitly tests the moderating effect on the predictor to mediator path (i.e., path a). An index of moderated mediation was used to test the significance of the moderated mediation or the difference of the indirect effects for the COVID-19 diagnosis groups. Significant effects are supported by the absence of zero within the confidence intervals.

To investigate whether medical providers report a higher level of current autonomic reactivity and if their increased autonomic reactivity relates to more MH difficulties, various analyses were run. First, chi square analyses evaluated whether the individuals diagnosed with COVID-19 were more likely to be providing medical care to COVID-19 patients. Next, ANOVA analyses explored the contributions and potential interaction of medical provider role and COVID-19 diagnosis on levels of current autonomic reactivity while considering age. Moderation analyses with age entered as a covariate investigated whether medical provider role moderated that relationship between current autonomic reactivity and emotional distress. Lastly, binary logistic regression analyses examined the contributions of medical provider role and COVID-19 diagnosis in predicting individuals who score above or below the clinical cutoff for PTSD.

## Results

### Participants

Participants (*N* = 1,638; 61% female; Age *M* = 46.80, *SD* =16.29, range 18–88 years old) were individuals living in the US that either reported no prior diagnosis or physical symptoms related to COVID-19 (*n* = 1,540) or having been diagnosed with COVID-19 (*n* = 98) currently (*n* = 61) or previously (*n* = 37). We found that 47.1% of the participants previously diagnosed with COVID-19 reported currently having symptoms that could be related to COVID-19, whereas 52.9 of participants that reported currently having COVID-19 reported currently having symptoms that could be related to COVID-19.The participants infected with COVID-19 were younger (*M* = 37.98, *SD* = 12.51) than those not infected by COVID-19 (*M* = 47.37, *SD* = 16.35), *F*_(1,1586)_ = 30.53, *p* <0.001; eta square = 0.019. The groups did not differ with regard to educational level or income.

#### Aim 1: Assess Differences in Current Autonomic Reactivity and Prior Mental Health Histories in Individuals Infected or Not Infected by COVID-19

As reported in Table 1, the COVID-19 diagnosis and no COVID-19 diagnosis groups differed in terms of level of their current levels of autonomic reactivity and the distributions within the groups are virtually non-overlapping. When focusing only on the 98 participants diagnosed with COVID-19, the 47 participants currently infected reported experiencing more autonomic reactivity (*M* = 70.01, *SD* = 11.13) than the previously infected (*M* = 64.14, *SD* = 13.60), *F*_(1,95)_ = 5.44, *p* =0.022; η^2^ =0.05. However, as evident by the eta square coefficients, the magnitude of these effects was noticeably smaller than the COVID-19/no COVID-19 contrasts.

**Table 1:** Vulnerability factors by COVID diagnosis groups.

	**COVID diagnosis (*n* = 98)**	**No COVID diagnosis (*n* = 1,540)**	***F*** **or** ***X**^**2**^*	***η2***
	***M (SD)***	***M (SD)***		
**Autonomic reactivity**	67.02 (12.73)	46.47 (9.42)	*F* = 417.09^***^	0.20
**Mental health history**				
Prior psychiatric diagnosis	0.98 (0.82)	0.77 (1.04)	*F* = *3.78*	0.00
Depression diagnosis	33.7%	24.2%	*X2* = 4.48^*^	0.00
Anxiety diagnosis	21.4%	26.8%	*X2* = 1.34	0.00
PTSD diagnosis	14.3%	14.5%	*X2* = 0.00	0.00
Impact of MH adversities	30.99 (14.47)	12.03 (10.08)	*F* = 303.42^***^	0.16
**Medical health history**				
Prior COVID medical risks	50.0%	22.2%	*X2* = 39.16^***^	0.05
Moderate to severe asthma	25.5%	7.3%	*X2* = 34.44^***^	0.02
Diabetes	28.6%	8.1%	*X2* = 45.52^***^	0.03
Impact of medical adversities	9.71 (4.90)	2.99 (3.68)	*F* = 294.24^***^	0.15

* *p < 0.05,*

** p < 0.01,

*** *p < 0.001*.

Table 1 also shows that the COVID-19 diagnosis and no COVID-19 diagnosis groups differed in their likelihood of previously being diagnosed with depression and a medical disorder that increases COVID-19 risk. While these differences are significant, the most striking differences are with regard to how impacted they were by their prior MH and medical adversities. Similar to current autonomic reactivity, the distributions within the groups are virtually non-overlapping.

**Figure 1:**
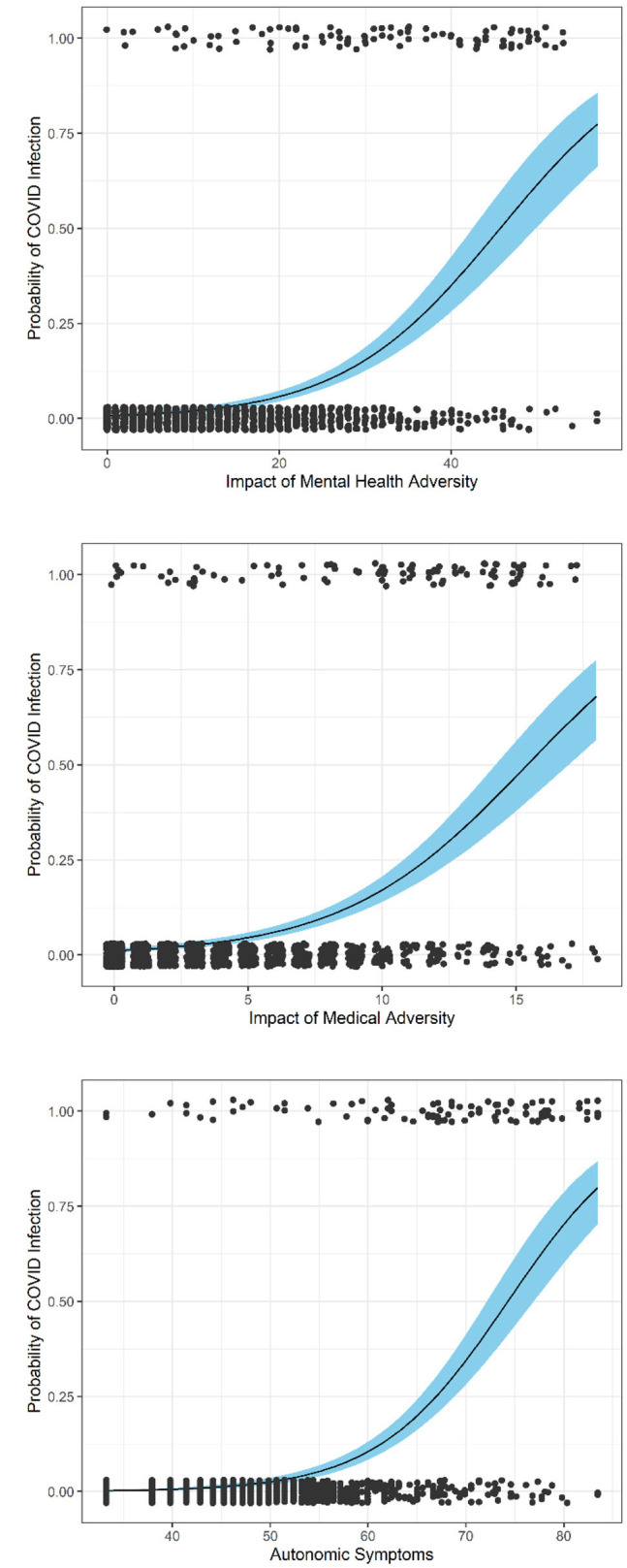
ROC models.

Thus, we investigated the relationship between reported impact of prior adversity, current autonomic reactivity, and likelihood being infected with COVID-19. Figure 1 shows ROC curves for current autonomic reactivity, prior MH adversity, and prior medical adversity on the probability of COVID-19 diagnosis. The probability of COVID-19 infection dramatically increases as scores increase for prior MH and medical adversity and for current autonomic reactivity.

#### Aim 2: Investigate Whether Individuals More Impacted by Their Prior MH and Medical Health Adversities Report Experiencing Higher Levels of Current Autonomic Reactivity

Linear regression analyses evaluated how prior MH and medical adversity is associated with current autonomic reactivity. The combination of variables accounted for 57.9% of variance for the COVID-19 diagnosis group, *F*_(2,94)_ = 64.66, *p* < 0.001, with prior medical adversities being a stronger predictor than prior MH adversities (*B* =0.49, *p* < 0.001 and *B* =0.32, *p* = 0.004). In contrast, for the no COVID-19 diagnosis group, the combination of variables only accounted for 24% of the variance, *F*_(2,1529)_ = 247.20, *p* < 0.001, with prior MH adversities being a stronger predictor than prior medical adversities (*B* =0.34, *p* < 0.001 and *B* =0.22, *p* < 0.001).

#### Aim 3: Determine Whether COVID-19 Diagnosis Interacts With Prior Adversity to Impact Current Autonomic Reactivity

Table 2 reports the results of two models that used hierarchical linear regression analyses to predict autonomic reactivity. For model 1, the hierarchical regression analyses determined that all three predictors (MH adversity, COVID-19 diagnosis, and interaction of MH adversity and COVID-19 diagnosis) entered in step 1 significantly predicted current autonomic reactivity and account for 39% of the variance. Although the inclusion of prior medical adversity in step 2 significantly increased the variance accounted for to 42%, it did not decrease the impact of the variables found to be significant in step 1.

**Table 2:** Results of stepwise linear regression analyses predicting autonomic reactivity from prior adversity and COVID-19 diagnosis.

**Predictors**	**Step 1**			**Step 2**		
	**Beta**	***t***	***p***	**Beta**	***t***	***p***
**Model 1**						
(Constant)		228.45	0.000		234.42	0.000
MH adversity	0.25	3.21	0.001	0.16	2.16	0.031
COVID-19 diagnosis	0.14	3.20	0.001	0.13	2.98	0.003
Interaction of MH adversity and COVID-19 diagnosis	0.30	3.03	0.002	0.24	2.44	0.015
Medical adversity				0.23	9.34	< 0.001
	*F*_(3,1625)_ = 350.47, *p* < 0.001; *R^2^* = 0.39			*F*_(4,1624)_ = 298.60, *p* < 0.001; *R^2^* = 0.42		
**Model 2**						
(Constant)		223.49	0.000		234.86	0.000
Medical adversity	0.05	0.66	0.509	−0.02	−26	0.794
COVID-19 diagnosis	0.10	2.42	0.016	0.10	2.43	0.015
Interaction of medical adversity and COVID-19 DIAGNOSIS	0.48	4.70	< 0.001	0.34	3.47	< 0.001
MH adversity				0.33	13.05	< 0.001
	*F*_(3,1625)_ = 312.29, *p* < 0.001; *R^2^* = 0.37			*F*_(4,1624)_ = 301.23, *p* < 0.001; *R^2^* = 0.43		

*Beta coefficients are standardized and analyses were run with Z scores to facilitate comparisons among the variables*.

Model 2 determined that the individual and combined impact of medical adversity and COVID-19 diagnosis accounted for 37% of the variance in autonomic reactivity and that the significant predictors were COVID-19 diagnosis and the interaction of medical adversity and COVID-19 diagnosis. Although the inclusion of prior MH adversity in step 2 significantly increased the variance accounted to 43%, it did not decrease the impact of the variables found to be significant step 1.

#### Aim 4: Determine Whether Increased Current Autonomic Reactivity Mediates the Relationship Between Prior Adversity and Current Mental Health Difficulties

As reported in Table 3, compared to those not infected with COVID-19, those infected with COVID-19 reported currently experiencing more emotional distress, greater mindfulness difficulties, and more posttraumatic stress symptoms, and were more likely to score above the clinical cutoff for PTSD and depression. With considerably large effect sizes, the greatest difference between the COVID-19 diagnosis groups was with regard to their level of emotional distress and posttraumatic stress symptomatology.

**Table 3:** Current mental health by COVID diagnosis groups.

	**COVID diagnosis**	**No diagnosis or symptoms**	***F*** **or** ***X**^**2**^*	***η2***
	***M (SD)***	***M (SD)***		
**Total scores**			F	
Emotional distress	40.57 (12.13)	24.66 (10.06)	224.46^***^	0.12
Mindfulness difficulties	3.61 (0.91)	2.79 (0.91)	74.47^***^	0.04
Posttraumatic stress	57.29 (16.93)	33.53 (15.09)	222.91^***^	0.12
Re-experiencing	3.49 (1.89)	1.09 (1.70)	178.78^***^	0.10
Avoidance	5.27 (2.22)	1.96 (2.23)	201.89^***^	0.11
Hyperarousal	3.89 (1.65)	1.62 (1.75)	153.07^***^	0.09
**Above clinical cutoff**			X^2^	
PTSD	75.3%	23.0%	129.31^***^	0.08
Depression symptoms	67.3%	25.5%	80.28^***^	0.05

* p < 0.05,

** p < 0.01,

*** *p < 0.001*.

**Figure 2:**
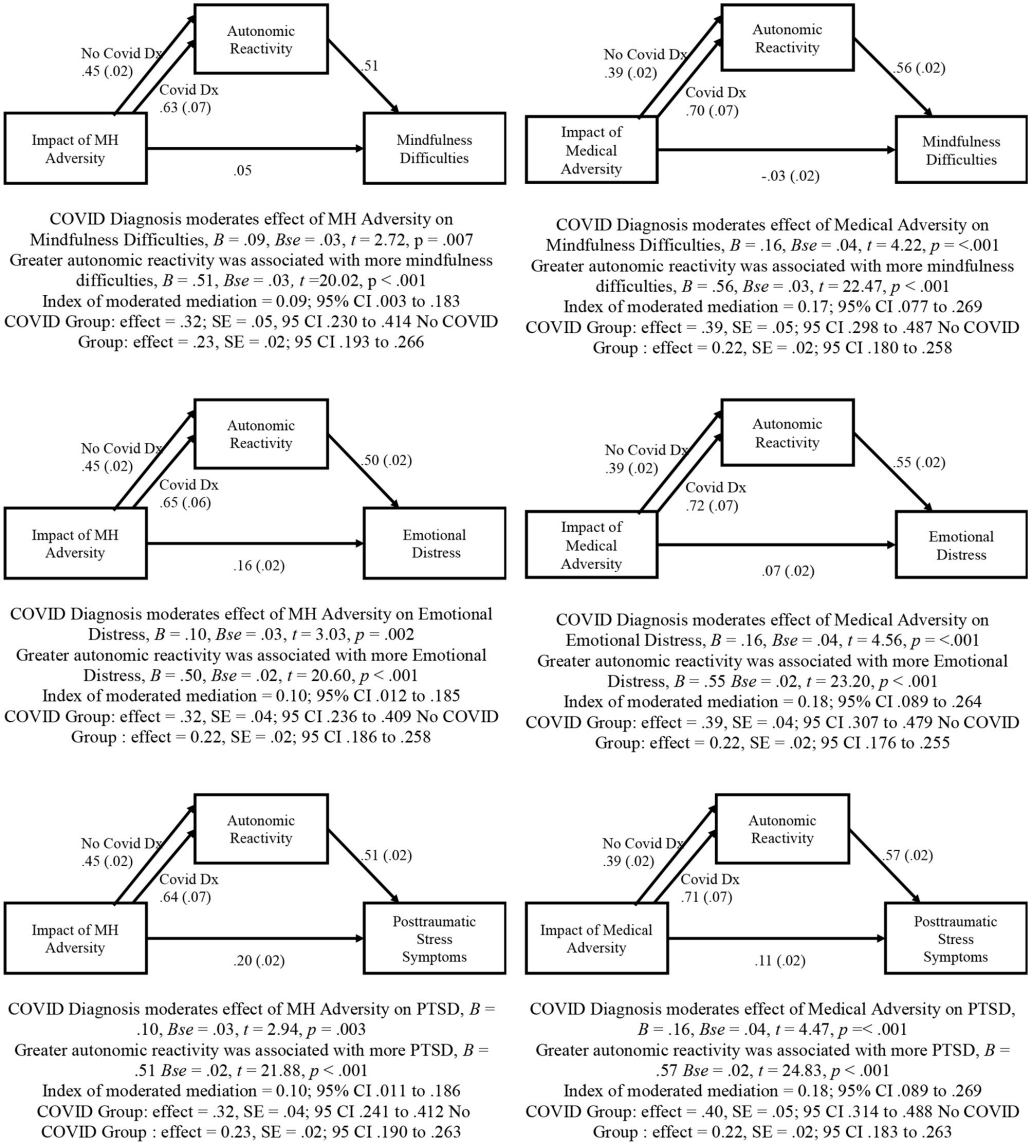
Moderated mediation models testing relationship among prior adversity, current autonomic reactivity, and current mental health difficulties.

**Figure 3:**
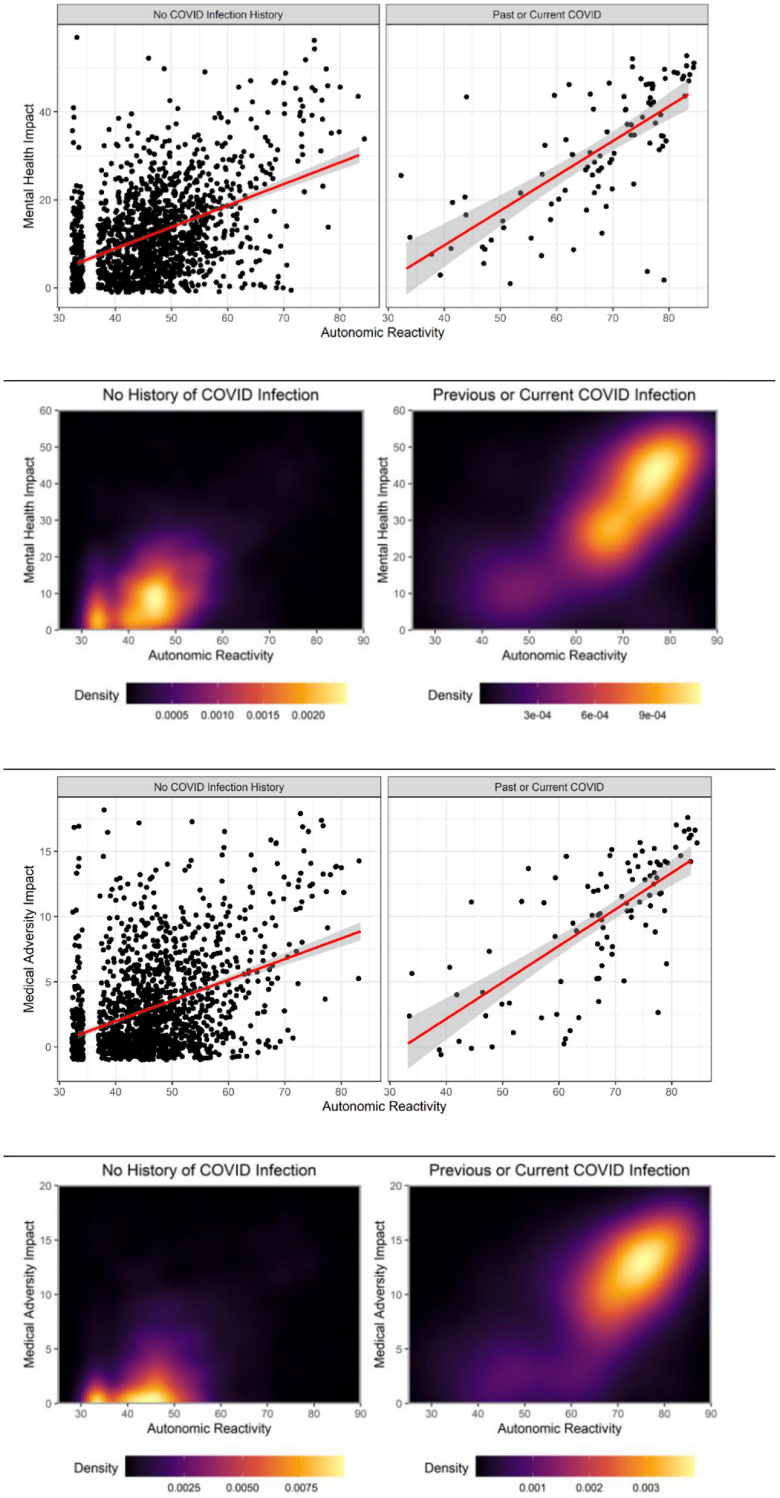
COVID-19 diagnosis moderates relationship between adversity and autonomic reactivity.

The hypothesized moderated mediation model was tested using the PROCESS macro model number 7. As reported in Figure 2, COVID-19 diagnosis moderated the effect of both prior MH and medical adversity on current MH difficulties (i.e., mindfulness difficulties, emotional distress, and posttraumatic stress). Zero is not within the CI in any of the models, indicating that COVID-19 diagnosis significantly moderates the direct effects of prior MH and medical adversity on current autonomic reactivity. These significant moderation results are displayed in Figure 3 *via* density plots and heat maps that are useful for visualizing areas where observations are more common.

Separate analyses indicated that current autonomic reactivity mediated a large percentage (between 58.9 and 85.2%) of the relationship between prior MH and medical adversity and current MH difficulties. However, as presented in all models in Figure 2, the moderated mediation results indicated that the conditional indirect effect was stronger in those diagnosed with COVID-19 and weaker in those without COVID-19 diagnosis.

#### Aim 5: Investigate Whether Medical Providers Report Higher Level of Current Autonomic Reactivity and if Their Increased Autonomic Reactivity Relates to More MH Difficulties

The participants who had been diagnosed with COVID-19 were more likely to be providing medical care to COVID-19 patients, $X_{\left(1,\text{n}=1,638\right)}^{2}$ = 164.35. Specifically, 34.3% of these medical providers were infected with COVID-19, whereas only 4.0% of the general population. Because of the higher rates of COVID-19 infection, the medical providers appeared to have a higher probability of having higher levels of current autonomic reactivity. ANOVA analyses indicated both COVID-19 diagnosis, *F*_(1,1635)_ = 339.17, *p* <0.001, and medical provider role, *F*_(1,1635)_ = 10.14, *p* =0.001, were significant predictors of current autonomic reactivity (Both Risks *M* = 69.27, *SD* = 11.41; Only COVID-19 Diagnosis *M* = 65.65, *SD* = 13.37; Only Medical Provider *M* = 49.41, *SD* = 12.74; No Risks = 46.33, *SD* = 9.12). There was an incremental increase in levels of current autonomic reactivity from the individual with both risks to the individuals with no risk factors. These differences remained when taking into account the significant age differences between the medical providers and the general population (medical providers *M* = 39.13, *SD* = 12.77 and general population *M* = 47.34, *SD* = 16.38), *F*_(1,1586)_ = 25.03, *p* < 0.001.

Moderation analyses with age entered as a covariate indicated that the medical provider role moderated the relationship between current autonomic reactivity and emotional distress, *F*_(1,1583)_ = 5.45, *p* = 0.020. Similar moderation results were found for overall posttraumatic stress symptoms, *F*_(1,1582)_ = 3.98, *p* = 0.046, and the components of re-experiencing, *F*_(1,1582)_ = 4.19, *p* =0.041; and avoidance, *F*_(1,1582)_ = 4.03, *p* = 0.045. The medical providers exhibited increased symptom severity as their levels of current autonomic reactivity increased. Although medical providers reported higher levels of hyperarousal and greater mindfulness difficulties, the effects were not influenced by their current autonomic reactivity.

In addition, binary logistic regression analyses indicated that both COVID-19 diagnosis (OR = 3.39, *p* < 0.001, 95% CI 2.55–4.52) and medical provider role (OR = 1.76, *p* = 0.009, 95% CI 1.15–2.68) significantly increased risk of scoring above the clinical cutoff for PTSD. Similarly, both having COVID-19 diagnosis (OR = 2.46, *p* < 0.001, 95% CI 1.90–3.18) and medical provider role (OR = 1.90, *p* = 0.002, 95% CI 1.26–2.85) significantly increased risk of scoring above the clinical cutoff for depression.

## Discussion

The current study investigates whether individuals infected with COVID-19 are experiencing higher levels of self-reported current autonomic reactivity and more MH difficulties, and whether their difficulties relate to their prior impact of MH and medical adversities. We also explored whether medical providers, who were caring for COVID-19 patients and had higher rates of COVID-19 infection, were having more MH difficulties than the general population. We found that participants infected with COVID-19 did report higher levels of current autonomic reactivity and more MH difficulties as well as being more impacted by prior MH and medical adversity. COVID-19 diagnosis moderated the effect of both prior MH and medical adversity on current autonomic reactivity and MH difficulties. Current autonomic reactivity also mediated the relationship between prior adversities and current MH difficulties. However, the effect was stronger in those also diagnosed with COVID-19. It was found that the medical provider role was associated with increased levels of current autonomic reactivity, especially for providers diagnosed with COVID-19.

Consistent with polyvagal theory, we conceptualized the current pandemic as a stressful event that may lead to increased autonomic reactivity that relates to a retuning of the ANS. Stress leads to defense responses that increase sympathetic activation and bias neuroception toward the detection of threat cues, while becoming less sensitive to the detection of safety cues (2, 3). Consistent with the limited research investigating autonomic regulation difficulties in individuals diagnosed with COVID-19 (4, 5), we found that these individuals had significantly higher levels of self-reported current autonomic reactivity than individuals in the general populations and other US samples [e.g., (34)]. Since COVID-19 infection is both emotionally and medically traumatic, it leads one to speculate there may be some vulnerability to autonomic dysregulation. Considering prior research investigating the relation between the immune system and ANS particularly for MS disease activity (45), it is possible ANS dysregulation could influence immune response and accompany a COVID-19 infection in similar ways.

We also found that the levels of current autonomic reactivity were higher in those diagnosed with COVID-19 at the time of data collection than those previously diagnosed with COVID-19. Although it is encouraging that the rates appear to have dropped over time, it is concerning that the individuals previously diagnosed had higher levels than those not diagnosed with COVID-19, which is consistent with prior findings that changes in the ANS may persist after the infection has dissipated (6).

Due to the stress of the risk of infection during the pandemic, the fear of significant morbidity due to becoming infected, and social isolation due to quarantining, we hypothesized that individuals diagnosed with COVID-19 would have more MH difficulties. Consistent with the prior research (7–14) we found that individuals diagnosed with COVID-19 reported higher levels of current MH difficulties. Specifically, we found higher levels of emotional distress on a measure designed to tap the symptoms of distress identified by the CDC which includes items related to anxiety and depression. We also found that COVID-19 survivors were experiencing more mindfulness difficulties, which is consistent with the clinical impressions of brain fog (16) and prior research findings suggesting that changes in brain function may occur due to differences in gray matter (15). Lastly, we found that these individuals were experiencing more posttraumatic stress symptoms, including re-experiencing, avoidance, and hyperarousal. The latter symptoms relate directly to the reported high level of current autonomic reactivity, and accompanying difficulty of feeling safe as would be predicted by polyvagal theory.

Consistent with polyvagal theory, we also investigated whether individuals who were more impacted by prior MH and medical adversities may be more vulnerable to and impacted by the COVID-19 infection. Rather than asking about the occurrence of an adverse event, we focused on the individual's perception of how impacted they were by their prior MH and medical adversities. This was important as we believed that those more impacted are likely to have experienced more frequent/severe events that potentially alter their ANS and lead to vulnerability to developing disease and experiencing more significant effects.

As hypothesized, we found that individuals reporting they were more impacted by their prior MH and medical adversities reported higher levels of current autonomic reactivity. We also observed that these variables accounted for 58% of the variance in current autonomic reactivity in the individuals diagnosed with COVID-19. As would be expected, their autonomic reactivity was more affected by their medical adversity than their MH adversity—although both were important predictors. In contrast, prior MH adversity was more important than prior medical adversity in predicting current autonomic reactivity in individuals not diagnosed with COVID-19. Thus, our results suggested that both MH and medical adversity may impact current autonomic reactivity.

We were able to demonstrate that COVID-19 diagnosis may impact the relationship between prior MH and medical adversity and current autonomic reactivity through hierarchical regression and moderated mediation analyses. We also found that COVID-19 diagnosis may moderate the effect of MH and medical adversity on the current MH difficulties of increased emotional distress, mindfulness difficulties, and posttraumatic stress symptoms. Specifically, we found that individuals infected with COVID-19 had higher levels of current autonomic reactivity and were more likely to exhibit an increase in autonomic reactivity as their adversity impact scores increased. Additionally, we found that current autonomic reactivity mediated a large percentage (between 58.9 and 85.2%) of the relationship between prior MH and medical adversity and current MH difficulties, with the indirect effect being stronger for individuals diagnosed with COVID-19 than in those without a COVID-19 diagnosis. Although no definitive statements can be made because of the cross-sectional design, it is possible that the previously observed connection between prior adversity, current autonomic reactivity, and current MH difficulties (1) may be exacerbated in individuals diagnosed with COVID-19. It is not clear if this is because of the virus or the stress associated with the COVID-19 diagnosis.

Consistent with prior statistics (30), we found that 34% of the medical providers had been diagnosed with COVID-19, a rate which was considerably higher than the 4% found in our general population. We also found that both risk factors (i.e., COVID diagnosis and medical provider role) were significant predictors of current autonomic reactivity and that there was an incremental increase in levels of autonomic reactivity from the individuals with no risks to the individuals with both risk factors. Not only was there an interactive effect, the medical provider role moderated the relationship between current autonomic reactivity and levels of emotional distress and posttraumatic stress symptoms, with the medical providers exhibiting greater symptom severity as their levels of autonomic reactivity increased. In addition, we found that both COVID-19 diagnosis and medical provider role increased risk of scoring above the clinical cutoff for PTSD and depression. Thus, our findings suggest that the reported MH difficulties (e.g., anxiety, depression, and PTSD) in medical workers previously diagnosed with COVID-19 (31) may be related to increased autonomic reactivity.

## Limitations and Future Directions

The potential implications of the current study need to be considered in the context of the study's limitations. With the cross-sectional study design, it is not possible to make any definitive statements regarding causality and to determine how much of the COVID-19 group differences are related to the virus or the stress associated with the COVID-19 diagnosis. It is also unknown whether the participants were including their COVID-19 diagnosis when answering the question about a life-threatening illness that is part of the six items that made up the medical adversity scale.

Additionally, the sample included a higher percentage of females which research suggests are more likely to report mental health symptoms (46). Despite the large sample size (*N* = 1,638), only 98 individuals reported having COVID-19 and there was no confirmation of diagnosis. Although not a large group, those diagnosed with COVID-19 did not differ from the general population in terms of demographic characteristics, which could have impacted other factor assessed in our study.

Because many of the participants that had COVID-19 were medical providers, we addressed this limitation by exploring the combined impact of these factors. Future research should incorporate a confirmation of COVID-19 diagnosis and consider the timing and severity of illness as there were small differences in our sample between those who had COVID-19 at the time of data collection and those who had been previously diagnosed.

Due to a need to quickly understand how individuals are coping with the pandemic and the data collection limitations related to the COVID-19 pandemic, current autonomic reactivity could only be collected *via* self-report. Although the BPQ-SF appears to be an appropriate measure of autonomic reactivity as it has high convergent validity with similar measures and consistency across samples (34), it is unclear whether the self-reports reflect autonomic state reactivity prior to the pandemic or is a sensitive index of the individual autonomic reaction to the pandemic. Future research should explore objective measurements of autonomic reactivity prior to, during, and following COVID-19 infection.

It is also important to consider the limitations related to the ATES, which is a new measure with limited psychometric information. However, the negatives of this instrument are outweighed by its positives, as it asks about the perceived impact of a range of traumatic experiences. Rather than simply documenting whether traumatic experience occurred, the ATES indirectly assesses the frequency and severity of adversity/trauma. Lastly, the measure of emotional distress was created to tap symptoms of distress as identified by the CDC. Thus, it asked questions related to shock about the situations induced by the pandemic and primarily focused on symptoms of anxiety and depression. Although this measure was found to be internally consistent, it may have been better to use established measures of anxiety or depression. However, it is important to note that the CDC emotional distress measure directly relates to the overarching large-scale crisis of the pandemic, which may encourage more nuanced responses than a standardized measure.

## Conclusion and Implications

Our results suggest that individuals diagnosed with COVID-19, particularly medical providers, may have increased levels of current autonomic reactivity that is associated with their prior MH and medical adversities and current MH difficulties. Our findings are consistent with polyvagal theory and prior research suggesting autonomic dysregulation (5, 6) and poorer mental health outcomes in COVID-19 survivors (7–14), and are unique in indicating that the combination of the COVID-19 diagnosis and medical provider role could lead to more detrimental effects.

Our results suggest an important avenue for clinical treatment may lie in interventions that focus on both the body and mind and that COVID-19 survivors and their medical providers should be provided with somatic-focused interventions and cognitive strategies that will retune their potentially dysregulated ANS. Prior research suggests bottom-up approaches to therapy helps individuals to connect with their bodies and their feelings, thus teaching them to calm their physiology (47). Improvements in regulation documented in interventions focused on yoga (48) and mindfulness body scan mediation (49) suggest that body focused interventions have tremendous potential to be helpful to populations at risk for increased autonomic reactivity by way of COVID-19 diagnosis, medical provider status, or both. These body-focused interventions may benefit from addressing spirituality, which by encouraging transcendence, connection, wholeness, and compassion (50), fosters resilience (50) and is associated with reductions in stress (51) and improvements in physical and mental wellbeing (50, 51). Previous studies have found spirituality has been associated with more hopefulness and less fear, worry, and sadness in the midst of the COVID-19 pandemic (50).

Interventions should be implemented in the workplace to encourage resilience and psychological wellbeing through the employment-related services and social/emotional support (52). This is a promising avenue for individuals working in healthcare because prior research shows that healthcare providers who do not feel supported by their leadership in the workplace are more likely to experience exhaustion and disengagement (29). Thus, integrating interventions into their workplace environments may alleviate some of the stressors that contribute to dysregulation, which is essential when considering the vital role that healthcare providers play during this time.

## Data Availability Statement

The raw data supporting the conclusions of this article will be made available by the authors, without undue reservation.

## Ethics Statement

The studies involving human participants were reviewed and approved by Indiana University Institutional Review Board. The patients/participants provided their written informed consent to participate in this study.

## Author Contributions

LD developed study, analyzed data, and wrote all sections of manuscript. SC and SP developed study, reviewed analyses, and reviewed manuscript edits. JK developed study, collected and reviewed data, made figures, and reviewed analyses and versions of manuscript. KL and NB wrote sections of manuscript and edited final version. AB wrote sections of manuscript. EN collected and reviewed data. All authors contributed to the article and approved the submitted version.

## Funding

Funding in support of this work was provided by the Dillon Foundation and the United States Association of Body Psychotherapy.

## Conflict of Interest

The authors declare that the research was conducted in the absence of any commercial or financial relationships that could be construed as a potential conflict of interest.

## Publisher's Note

All claims expressed in this article are solely those of the authors and do not necessarily represent those of their affiliated organizations, or those of the publisher, the editors and the reviewers. Any product that may be evaluated in this article, or claim that may be made by its manufacturer, is not guaranteed or endorsed by the publisher.

## References

[1] Kolacz J Dale LP Nix EJ Roath OK Lewis GF Porges SW. Adversity history predicts self-reported autonomic reactivity and mental health in US residents during the COVID-19 pandemic. Front Psychiatry. (2020) 11:e577728. 10.3389/fpsyt.2020.577728 33192715 PMC7653174

[2] Porges SW. The COVID-19 pandemic is a paradoxical challenge to our nervous system: a polyvagal perspective. Clin Neuropsychiatry. (2020) 17:135–8. 10.36131/CN20200220 34908984 PMC8629069

[3] Porges SW. The polyvagal perspective. Biol Psychol. (2007) 74:116–43. 10.1016/j.biopsycho.2006.06.009 17049418 PMC1868418

[4] Becker RC. Autonomic dysfunction in SARS-COV-2 infection acute and long-term implications COVID-19 editor's page series. J Thromb Thrombolysis. (2021) 52:692–707. 10.1007/s11239-021-02549-6 34403043 PMC8367772

[5] Milovanovic B Djajic V Bajic D Djokovic A Krajnovic T Jovanovic S. Assessment of autonomic nervous system dysfunction in the early phase of infection with SARS-CoV-2 virus. Front Neurosci. (2021) 15:e640835. 10.3389/fnins.2021.640835 34234638 PMC8256172

[6] Buoite SA Furlanis G Frezza NA Valentinotti R Ajcevic M Manganotti P. Autonomic dysfunction in post-COVID patients with and without neurological symptoms: a prospective multidomain observational study. J Neurol. (2022) 269:587–96. 10.1007/s00415-021-10735-y 34386903 PMC8359764

[7] Cabrera MA Karamsetty L Simpson SA. Coronavirus and its implications for psychiatry: a rapid review of the early literature. Psychosomatics. (2020) 61:607–15. 10.1016/j.psym.2020.05.018 32943211 PMC7251405

[8] Cai X Hu X Ekumi IO Wang J An Y Li Z. Psychological distress and its correlates among COVID-19 survivors during early convalescence across age groups. Am J Geriatr Psychiatry. (2020) 28:1030–9. 10.1016/j.jagp.2020.07.003 32753338 PMC7347493

[9] Di Gennaro F Pizzol D Marotta C Antunes M Racalbuto V Veronese N. Coronavirus diseases (COVID-19) current status and future perspectives: a narrative review. Int J Environ Res Public Health. (2020) 17:2690. 10.3390/ijerph17082690 32295188 PMC7215977

[10] Hu Y Chen Y Zheng Y You C Tan J Hu L. Factors related to mental health of inpatients with COVID-19 in Wuhan, China. Brain Behav Immun. (2020) 89:587-93. 10.1016/j.bbi.2020.07.016 32681866 PMC7362867

[11] Liu D Baumeister RF Zhou Y. Mental health outcomes of coronavirus infection survivors: a rapid meta-analysis. J Psychiatr Res. (2021) 137:542–53. 10.1016/j.jpsychires.2020.10.015 33436263 PMC7576143

[12] Mazza MG Palladini M De Lorenzo R Magnaghi C Poletti S Furlan R. Persistent psychopathology and neurocognitive impairment in COVID-19 survivors: effect of inflammatory biomarkers at three-month follow-up. Brain Behav Immun. (2021) 94:138–47. 10.1016/j.bbi.2021.02.021 33639239 PMC7903920

[13] Vindegaard N Benros ME. COVID-19 pandemic and mental health consequences: systematic review of the current evidence. Brain Behav Immun. (2020) 89:531–42. 10.1016/j.bbi.2020.05.048 32485289 PMC7260522

[14] Yalçin I Can N Mançe Çalişir Ö Yalçin S Çolak B. Latent profile analysis of COVID-19 fear, depression, anxiety, stress, mindfulness, and resilience. Curr Psychol. (2022) 41:459-69. 10.1007/s12144-021-01667-x 33821112 PMC8012016

[15] Douaud G Lee S Alfaro-Almagro F Arthofer C Wang C Lange F. Brain imaging before and after COVID-19 in UK Biobank. medRxiv. (2021) 10.1101/2021.06.11.21258690 34189535 PMC8240690

[16] Graham EL Clark JR Orban ZS Lim PH Szymanski AL Taylor C. Persistent neurologic symptoms and cognitive dysfunction in non-hospitalized Covid-19 long haulers. Ann Clin Transl Neurol. (2021) 8:1073–85. 10.1002/acn3.51350 33755344 PMC8108421

[17] Kemp AH Quintana DS. The relationship between mental and physical health: insights from the study of heart rate variability. Int J Psychophysiol. (2013) 89:288–96. 10.1016/j.ijpsycho.2013.06.018 23797149

[18] Staud R. Heart rate variability as a biomarker of fibromyalgia syndrome. Fut Rheumatol. (2008) 3:475–83. 10.2217/17460816.3.5.475 19890437 PMC2772072

[19] Staud R. Autonomic dysfunction in fibromyalgia syndrome: postural orthostatic achycardia. Curr Rheumatol Rep. (2008) 10:463–6. 10.1007/s11926-008-0076-8 19007537

[20] Kenney MJ Ganta CK. Autonomic nervous system and immune system interactions. Compr Physiol. (2014) 4:1177–200. 10.1002/cphy.c130051 24944034 PMC4374437

[21] Yoo BB Mazmanian SK. The enteric network: Interactions between the immune and nervous systems of the gut. Immunity. (2017) 46:910–26. 10.1016/j.immuni.2017.05.011 28636959 PMC5551410

[22] Won E Kim YK. Stress, the autonomic nervous system, and the immune-kynurenine pathway in the etiology of depression. Curr Neuropharmacol. (2016) 14:665–73. 10.2174/1570159x14666151208113006 27640517 PMC5050399

[23] Habek M. Immune and autonomic nervous system interactions in multiple sclerosis: clinical implications. Clin Auton Res. (2019) 29:267–75. 10.1007/s10286-019-00605-z 30963343

[24] Muller AE Hafstad EV Himmels JPW Smedslund G Flottorp S Stensland SØ. The mental health impact of the covid-19 pandemic on healthcare workers, and interventions to help them: a rapid systematic review. Psychiatry Res. (2020) 293:113441. 10.1016/j.psychres.2020.113441 32898840 PMC7462563

[25] Chirico F Ferrari G Nucera G Szarpak L Crescenzo P Ilesanmi O. Prevalence of anxiety, depression, burnout syndrome, and mental health disorders among healthcare workers during the COVID-19 pandemic: a rapid umbrella review of systematic reviews. J Health Soc Sci. (2021) 6:209–20. 10.19204/2021/prvl7

[26] Ahmed F Zhao F Faraz NA Qin YJ. How inclusive leadership paves way for psychological well-being of employees during trauma and crisis: a three-wave longitudinal mediation study. J Adv Nurs. (2021) 77:819–31. 10.1111/jan.14637 33231300 PMC7753635

[27] Zhizhong W Koenig HG Yan T Jing W Mu S Hongyu L. Psychometric properties of the moral injury symptom scale among chinese health professionals during the COVID-19 pandemic. BMC Psychiatry. (2020) 20:e556. 10.1186/s12888-020-02954 33238949 PMC7686837

[28] Mosheva M Gross R Hertz PN Hasson OI Kaplan R Cleper R. The association between witnessing patient death and mental health outcomes in frontline COVID-19 healthcare workers. Depress Anxiety. (2021) 38:468–79. 10.1002/da.23140 33544405 PMC8014064

[29] Dale LP Cuffe SP Sambuco N Guastello AD Leon KG Nunez LV. Morally distressing experiences, moral injury, and burnout in Florida healthcare providers during the COVID-19 pandemic. Int J Environ Res Public Health. (2021) 18:12319. 10.3390/ijerph182312319 34886045 PMC8656473

[30] Nguyen LH Drew DA Graham MS Joshi AD Guo CG Ma W. Risk of COVID-19 among front-line health-care workers and the general community: a prospective cohort study. Lancet Public Health. (2020) 5:e475–83. 10.1016/S2468-2667(20)30164-X 32745512 PMC7491202

[31] Mohammadian Khonsari N Shafiee G Zandifar A Mohammad Poornami S Ejtahed HS Asayesh H. Comparison of psychological symptoms between infected and non-infected COVID-19 health care workers. BMC Psychiatry. (2021) 21:170. 10.1186/s12888-021-03173-7 33771122 PMC7995388

[32] Porges SW. Body Perception Questionnaire. Maryland, MD: Laboratory of Developmental Assessment, University of Maryland (1993).

[33] Kolacz J Holmes LG Porges SW. Body Perception Questionnaire (BPQ) Manual. Bloomington, IN. (2018).

[34] Cabrera A Kolacz J Pailhez G Bulbena-Cabre A Bulbena A Porges SW. Assessing body awareness and autonomic reactivity: factor structure and psychometric properties of the Body Perception Questionnaire-Short Form (BPQ-SF). Int J Methods Psychiatr Res. (2018) 27:e1596. 10.1002/mpr.1596 29193423 PMC6877116

[35] Kolacz J Chen X Nix EJ Roath OK Holmes LG Tokash C. Measuring autonomic symptoms with the body perception questionnaire short form (BPQ-SF): Factor analysis, derivation of U.S. adult normative values, and association with sensor-based physiological measures. medRxiv. (2022). 10.1101/2022.04.27.22274391

[36] Cerritelli F Galli M Consorti G D'Alessandro G Kolacz J Porges SW. Cross-cultural adaptation and psychometric properties of the Italian version of the body perception questionnaire. PLoS ONE. (2021) 16:e0251838. 10.1371/journal.pone.0251838 34043660 PMC8158925

[37] Dale LP Davidson C Kolacz J. Adverse Traumatic Experiences Scale. Jacksonville, FL. (2020).

[38] Brown KW Ryan RM. The benefits of being present: mindfulness and its role in psychological well-being. J Pers Soc Psychol. (2003) 84:822-48. 10.1037/0022-3514.84.4.822 12703651

[39] Weathers FW Huska JA Keane TM. PCL-C for DSM-IV. Boston: National Center for PTSD-Behavioral Science Division (1991).

[40] Wilkins KC Lang AJ Norman SB. Synthesis of the psychometric properties of the PTSD checklist (PCL) military, civilian, specific versions. Depress Anxiety. (2011) 28:596–606. 10.1002/da.20837 21681864 PMC3128669

[41] Ruggiero KJ Ben KD Scotti JR Rabalais AE. Psychometric properties of the PTSD checklist—civilian version. J Trauma Stress. (2003) 16:495–502. 10.1023/A:1025714729117 14584634

[42] Kroenke K Spitzer RL Williams JBW. The patient health questionnaire 2: validity of a two-item depression screener. Med Care. (2003) 41:1284–92. 10.1097/01.MLR.0000093487.78664.3C 14583691

[43] Spitzer RL Kroenke K. Williams JBW. Validation and utility of a self-report version of PRIME-MD: the PHQ 677 primary care study. JAMA. (1999) 282:1737–44. 10.1001/jama.282.18.1737 10568646

[44] Hayes AF. Introduction to Mediation, Moderation, and Conditional Process Analysis: A Regression-Based Approach. New York, NY: The Guilford Press (2013).

[45] Racosta JM Kimpinski K. Autonomic dysfunction, immune regulation and multiple sclerosis. Clin Auton Res. (2016) 26:23-31. 10.1007/s10286-015-0325-7 26691635

[46] Hubbard K Reohr P Tolcher L Downs A. Stress, mental health symptoms, and help-seeking in college students. Psi Chi J Psychol Res. (2018) 23:293–305. 10.24839/2325-7342.JN23.4.293

[47] Solomon EP Heide KM. The biology of trauma: implications for treatment. J Interpers Violence. (2005) 20:51–60. 10.1177/0886260504268119 15618561

[48] Goldstein MR Lewis GF Newman R Brown JM Bobashev G Kilpatrick L. Improvements in well-being and vagal tone following a yogic breathing based life skills wokshop in young adults: two open-trial pilot studies. Int J Yoga. (2016) 9:20–6. 10.4103/0973-6131.171718 26865767 PMC4728954

[49] Ditto B Eclache M Goldman N. Short-term autonomic and cardiovascular effects of mindfulness body scan meditation. Ann Behav Med. (2006) 32:227–34. 10.1207/s15324796abm3203_9 17107296

[50] Chirico F. Spirituality to cope with COVID-19 pandemic, climate change and future global challenges. J Health Soc Sci. (2021) 6:151–8. 10.19204/2021/sprt2

[51] Chirico F Nucera G. An Italian experience of spirituality from the Coronavirus pandemic. J Relig Health. (2020) 59:2193-95. 10.1007/s10943-020-01036-1 32424660 PMC7233189

[52] Chirico F Ferrari G. Role of the workplace in implementing mental health interventions for high-risk groups among the working age population after the COVID-19 pandemic. J Health Soc Sci. (2021) 6:145–50. 10.19204/2021/rlft1

